# Stress Induced Neuroplasticity and Mental Disorders

**DOI:** 10.1155/2017/9634501

**Published:** 2017-07-13

**Authors:** Fushun Wang, Fang Pan, Lee A. Shapiro, Jason H. Huang

**Affiliations:** ^1^School of Psychology, Nanjing University of Chinese Medicine, Nanjing 210023, China; ^2^Department of Medical Psychology, School of Basic Medical Sciences, Shandong University, Jinan 250012, China; ^3^Department of Surgery, Texas A&M College of Medicine, Temple, TX 76504, USA; ^4^Department of Neurosurgery, Baylor Scott & White Health, Temple, TX 76508, USA

Stress was introduced by Hans Selye as the general adaptation syndrome, which is a process under which the body confronts noxious agents. In the face of stress, the first reaction of the body is an alarm reaction, and the body prepares itself for “fight or flight.” And the second stage is adaptation of resistance, for the organism cannot sustain this motivated state too long. Finally, “due to wear and tear,” the body enters a third stage of exhaustion. Selye's brilliant ideas about stress helped an entirely new field to be forged and attracted thousands of researchers to work on the biological mechanism of stress. Stress was later extended to the field of psychology and redefined as the presence of acute or persistent physiological or psychological threats to the organism that result in significant strain on the body's compensatory systems, because anticipated thoughts about the stressful events can also induce stressful reactions. In addition, if the biological threat is easily avoidable, the body will not be stressed. Therefore, stressful reactions depend on the uncertain thought about the stressful events.

Lazarus proposed that stress depends on the thoughts and appraisals of the importance of the individual analysis of subjective appraisal of the situation. He distinguished two kinds of appraisals. The first appraisal is automatic, unreflective and unconscious, and fast activating, which is related to harm and threat, and induces fearful emotion to motivate avoidance and withdraw. The second is conscious and concerned with coping. In the face of threat, the organism was scared at first and showed fearful emotions. And then to cope with the threats, the organism collects energy in the body trying to “fight or flight” and shows angry emotions. Therefore, fear and anger usually come in a tandem at stressful events; fear is the scariness at the stressful events, and anger is coping with the threats. Lazarus suggested that fear and anger are hard to be detached and are two sides of the same coin. Similar to Selye's three stages of stress, Lazarus suggested another stage of reappraisal after coping with the situation. An individual employs two kinds of reappraisals: problem-focused and emotion-focused. If the organism can cope successfully with the stressful situation, the organism will get positive emotions and be happy. If the organism failed to cope with the situation, the organism would get negative emotions and be sad. Therefore, stressful event-induced emotions will go through a “fear-anger-joy-sadness” process, like a rainbow after a storm (Figure 1). If the organism cannot overcome the stressful events, like chronic stresses, some mental disorders will appear. The relation of the process with the emotional changes and mental disorders is shown in Figure 1.

The mechanisms whereby external stressors affect brain function have been the subject of extensive study over the past 60 years. Corticotropin-releasing hormone (CRH), which was named the stress hormone, has become the focus of the study. In both animals and humans, CRH can simulate norepinephrine (NE) synthesis and release. The central NE system innervates many brain areas, sympathetic nervous systems. CRH release also activates the hypothalamic-pituitary-adrenal (HPA) axis, which has been widely accepted as one of the central mechanisms involved in stress. The CRH induced release of ACTH (adrenocorticotropic hormone) and can in turn alter the function of the neural network by altering building blocks in the networks and by altering the integrative properties, and thus the behavioral or emotional changes. This neural plasticity underlying stress undoubtedly affects the brain function and may prompt functional alternations in mental disorders. Indeed, stress-induced neuroplasticity plays a critical role in almost all of the mental disorders, and stress has become a synonym for diverse terms of negative emotions, such as depression and anxiety. In this special issue, we collected papers on stress-induced neural plasticity and some neurological diseases. These reviews and experimental papers present the evidence that stress indeed affects long-term synaptic changes and induces many diseases such as depression and chronic pain.

In the review paper “Safety Needs Mediate Stressful Events Induced Mental Disorders,” Z. Zheng et al. reviewed recent papers about stress and proposed a new theory about human needs, which challenged the cornerstone theory of Maslow's famous theory of hierarchy of needs: safety needs come second to physiological needs. In this article, they propose that safety needs are more important and basic than physiological needs. They also probed into the neural basis of the stress system to be LC/NE system, and HPA system, which can be regarded as a “safety circuitry,” whose major behavior function is “fight or flight” and “fear and anger” emotions.

In the experimental paper “Shuyu Capsules Relieve Premenstrual Syndrome Depression by Reducing 5-HT_3A_R and 5-HT_3B_R Expression in the Rat Brain,” F. Li et al. reported a special treatment for the premenstrual depression. In this paper, the author reported that the premenstrual syndrome depression is characterized by changes in the subtypes of 5-HT receptors, especially the 5-HT_3_ receptors. In a special kind of rat depression model, they found that a kind of Chinese herb named Shuyu capsule can affect the expression levels of these receptors and can possibly be used to treat depression.

In the experimental paper “ATP Induces Disruption of Tight Junction Proteins via IL-1 Beta-Dependent MMP-9 Activation of Human Blood-Brain Barrier *In Vitro*,” F. Yang et al. reported that ATP released after blood-brain barrier impairments under brain traumatic stress affected P_2_X_7_ receptors and induced neural plasticity in the brain.

In the experimental paper “Activation of Sphingosine 1-Phosphate Receptor 1 Enhances Hippocampus Neurogenesis in a Rat Model of Traumatic Brain Injury: An Involvement of MEK/Erk Signaling Pathway,” Y. Ye et al. reported MEK/ERK signaling pathway in the neural plasticity under traumatic events.

In the review paper “The Role of Stress Regulation on Neural Plasticity in Pain Chronification,” the authors X. Li and L. Hu reviewed papers about stress and reported two pathways for stress: the faster pathway is the catecholamine, including norepinephrine and dopamine, priming the body into a kind of “fight or flight” state, and the slower process, including HPA changes. The neural plasticity under stress might be the substrate for chronic pain.

In the experimental paper “Profiling Proteins in the Hypothalamus and Hippocampus of a Rat Model of Premenstrual Syndrome Irritability,” M. Qiao et al. reported molecular changes in the hypothalamus and hippocampus under premenstrual depression, including Ulip2, tubulin, actin, interleukin, increased Kapp-beta binding motif phosphoprotein, and many other proteins. This paper will help understand the pathogenesis of premenstrual depression.

In the experimental paper “Neuroplastic Correlates in the mPFC Underlying the Impairment of Stress-Coping Ability and Cognitive Flexibility in Adult Rats Exposed to Chronic Mild Stress during Adolescence,” Y. Zhang et al. reported very interesting studies about influences of chronic mild stress in adolescence on cognition emotion and behavior and neurochemistry changes in the prefrontal cortex and suggested that the chronic stress-induced epigenetic changes might be the reason for the correlation between the emotional changes and neurochemistry changes.

In the experimental paper “A Combined Water Extract of Frankincense and Myrrh Alleviates Neuropathic Pain in Mice via Modulation of TRPV1,” D. Hu et al. reported some changes in TRPV1 channels under chronic pain, and they introduced a kind of Chinese herb which helps recovery from the chronic pain by modulation of TRPV1 channels.

In the experimental paper “Pathological Role of Peptidyl-Prolyl Isomerase Pin1 in the Disruption of Synaptic Plasticity in Alzheimer's Disease,” L. Xu et al. reported stress-induced loss of Pin1 protein activity enhances ubiquitin, which in turn induced synaptic loss and leads to Alzheimer's disease.

Collectively, these studies demonstrate that stress can induce many critical changes in the neural plasticity underlying many neurological diseases. We hope that this special issue will stimulate interests in the field of mechanisms of stress-inducing synaptic disorder and will help achieve a deeper understanding of the molecular mechanism of stress-induced disorders.

## Figures and Tables

**Figure 1:**
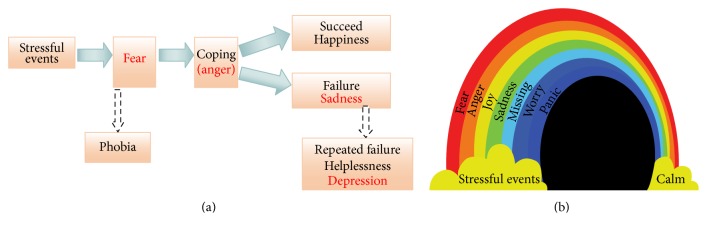
Stress-induced emotion flow and mental disorders. (a) At stressful events or threatening events, an organism will first be scared with the fear emotion. And to cope with the stressful events, the organism collects energy in the body to try to “fight or flight” with angry emotions. Fear is the scariness at the stressful events, and anger is coping with the stressful events. If the organism can cope successfully with the stressful situation, the organism will reappraise the situation and get positive emotions and be happy. If the organism failed to cope with the situation, the organism would get negative emotions and be sad. Stress-fear-response (anger)-consequences (happiness or sadness) constitute the emotion flow in our lives. (b) The emotion flow at stressful events is like a rainbow after a storm.

